# In situ simulation training in helicopter emergency medical services: feasible for on-call crews?

**DOI:** 10.1186/s41077-020-00126-0

**Published:** 2020-06-15

**Authors:** Per P. Bredmose, Jostein Hagemo, Jo Røislien, Doris Østergaard, Stephen Sollid

**Affiliations:** 1 Department of Research, Norwegian Air Ambulance Foundation, Drøbak, Norway; 2 Air Ambulance Department, Oslo University Hospital, Postboks 414, Sentrum, 0103 Oslo, Norway; 3 Faculty of Health Sciences, University of Stavanger, Stavanger, Norway; 4 Copenhagen Academy for Medical Education and Simulation, Capital Region of Denmark and University of Copenhagen, Copenhagen, Denmark

**Keywords:** Simulation, Prehospital, Air ambulance, Training, Education, In situ

## Abstract

Simulation-based training of emergency teams offers a safe learning environment in which training in the management of the critically ill patient can be planned and practiced without harming the patient. We developed a concept for in situ simulation that can be carried out during on-call time. The aim of this study is to investigate the feasibility of introducing in situ, simulation-based training for the on-call team on a busy helicopter emergency medical service (HEMS) base.

We carried out a one-year prospective study on simulation training during active duty at a busy Norwegian HEMS base, which has two helicopter crews on call 24/7. Training was conducted as low fidelity in situ simulation while the teams were on call. The training took place on or near the HEMS base. Eight scenarios were developed with learning objectives related to the mission profile of the base which includes primary missions for both medical and trauma patients of all ages, and interhospital transport of adults, children, and neonates. All scenarios included learning objectives for non-technical skills. A total of 44 simulations were carried out. Total median (quartiles) time consumption for on-call HEMS crew was 65 (59-73) min. Time for preparation of scenarios was 10 (5-11) min, time for simulations was 20 (19-26) min, cleaning up 7 (6-10) min, and debrief 35 (30-40) min. For all items on the questionnaire, the majority of respondents replied with the two most positive categories on the Likert scale. Our results demonstrate that in situ simulation training for on-call crews on a busy HEMS base is feasible with judicious investment of time and money. The participants were very positive about their experience and the impact of this type of training.

## Introduction

Physician staffed helicopter emergency medical services (HEMS) provide advanced prehospital critical care and are an integral part of many emergency medical services (EMS) worldwide. The provision of such care requires up-to-date knowledge and maintenance of certain skills. Clinical exposure to any particular presentation cannot be guaranteed, and it can be difficult to maintain clinical currency in a high workload HEMS system [1]. Delivering high-quality care also requires non-technical skills (NTS), which comprise cognitive skills (such as situational awareness and decision-making) and social skills (for example leadership, communication, and teamwork) [2]. These skills have for decades been a mandatory part of training programs in aviation. The HEMS pilot and crew members are explicitly trained in non-technical skills, but the full crew is rarely trained together [3]. Similarly, the pilot and HEMS crew member (HCM) are not trained in how to assist the physician in providing care for the patient.

Simulation-based training of in-hospital emergency teams has gained popularity as it provides a safe learning environment in which training in the management of the critically ill patient can be planned and practiced without harming the patient [4]. The content, volume, and frequency of training can be adapted and portioned to suit individual needs [5]. Recent findings suggest that brief, low fidelity, but high-frequency simulation training can be effective for training in newborn resuscitation, as well as for work in the operating theater [6, 7]. Simulation training can be a cost-effective way of maintaining skills and competence, [8]. One way of reducing the cost further is to conduct the training in the workplace, as in situ training [9]. Some leading HEMS services have implemented in situ simulation programs, although not specifically targeted at the crew on call [10]. In medical education, the focus is now on workplace-based learning [9]. We speculated, if introducing a program of simulation-based training, to be carried out when the HEMS crew is waiting for a new mission could be feasible and reduce costs associated with training [10].

The aim of this study is to investigate the feasibility of introducing in situ, simulation-based training for the on-call team on a busy HEMS base. We evaluated the time needed to prepare and carry out the training, and the participants’ self-reported reactions toward this type of training.

## Methods

### Location for training

The study was carried out as a prospective study at the HEMS base of Oslo University Hospital (OUH) in Norway, from January 1, 2012 to December 31, 2012 [11]. The base operates two helicopters 24/7 covering a population of approximately two million people. In 2012, the service performed 2577 missions [11]. The case mix includes primary trauma and medical missions; inter-hospital intensive care transfer of ventilated patients; specialized transfer of patients on organ support (e.g., extracorporeal membrane oxygenation (ECMO)) and sick neonatal patients in incubators.

### HEMS crew composition

Each HEMS helicopter in Norway is staffed by a three-person crew consisting of a physician (anesthesiologist), a HEMS crew resides member (HCM) and a pilot. The team on the HEMS base throughout the shift. The pilots are highly experienced pilots, with a civil and/or military aviation background. The HCM are emergency medical technicians, paramedics, or nurses, who have received extensive additional training in rescue techniques and aviation theory in order to be able to assist the pilot in navigation and planning of flights. Pilots and HCM are required to do bi-annual simulation training and tests in a flight simulator. There are no formal requirements for simulation training for doctors, who therefore rely on the availability of simulation-based training at their hospital, which is rarely mandatory and often irregular. All crew members are required to do regular training on fixed rope rescue operations and an annual aeromedical crew resource management course. HCMs and pilots are on call for 1 week at a time and the physician for 48 or 72 h.

### In situ simulation-based training concept

Simulation-based training builds on social constructivist theories to guide participants through a cycle of learning involving exposure to a scenario and a debriefing with discussion and reflection [12].

The on-call crews were offered the opportunity to train on a specific weekday between 9 am and 4 pm throughout the study period, except during public and school holidays, when the HEMS teams have too many missions to make training feasible. Training was voluntary and only took place if the on-call crews were rested: i.e., not in a mandatory rest period due to high duty load (> 14 h duty within the last 24 h) or by subjective evaluation by the crew themselves. No crew members were able to change their working schedules to either opt in or out of the simulation. Due to variation in the composition of the crews, individuals participated in a varying number of simulations.

Eight patient scenarios were developed by the main facilitator (PB) in consultation with a physician on the base. The scenarios were chosen to cover a wide range of topics relevant to the service, with a focus on current guidelines and best practice (Table 1) and developed in accordance with existing standard operating procedures if available. In all scenarios, the focus was medical treatment and correct use of equipment, and the use of non-technical skills and optimal crew resource management. The facilitator observed the actions of the crew and made the manikin respond accordingly. This form of tailoring the simulation was used to maximize the crew’s be immersed and integrated into the scenario.

**Table 1:** Simulation scenarios and medical learning objectives

No.	Scenario	Scenario content	Learning objectives	Clinical vignette
1	RSI 1	Subarachnoid bleeding	To safely conduct an RSI in an adult patient with subarachnoid hemorrhage with focus on induction of anesthesia and neurocritical care, team organization, and situational control	Age: 62 years GCS: E2V2M4 = 8 BP: 198/105 HR: 85
2	RSI 2	Convulsions in an adult patient	To handle a difficult airway during RSI of a fitting patient and apply neuroprotective treatment early in the prehospital phase	Age: 25 years GCS: E1V1M2 = 4 BP: 135/88 HR: 105
3	Trauma 1	Fall from height	To perform a rapid sequence induction in a trauma patient and apply early neuroprotective treatment whilst managing scene safety for the whole team	Age: 18 years GCS: E1V2M4 = 7 BP: 110/82 HR: 75
4	Trauma 2	Difficult access to a trauma patient	To control a trauma scene with a patient with an entrapped extremity and provide proper analgesia perform a safe trauma RSI including optimal use of the team	Age: 37 years GCS: E4V5M6 = 15 BP: 125/80 HR: 95
5	Interhospital retrieval	An ICU patient on CPAP	To undertake the interhospital transfer of a patient on CPAP and step up to BiPAP as required	Age: 70 years GCS: E3V5M6 = 14 BP: 105/75 HR: 84
6	Hypothermia	Continuous cardiopulmonary resuscitation during transportation	To initiate advanced life support and apply a mechanical chest compression device to continue CPR en-route to hospital	Age: 42 years GCS: E1V1M1 = 3 BP: Not recordable HR: 24 Temperature: 18.5 °C
7	Intoxication	Cocaine intoxication	To treat a severely intoxicated patient whilst addressing social concerns about the patient’s wellbeing	Age: 21 years GCS: E3V3M4 = 10 BP: 190/110 HR: 135
8	Paediatric RSI	A child with convulsions	To follow the algorithm for anticonvulsant treatment in a child and perform a safe pediatric RSI as a team	Age: 5 years GCS: E1V1M1 = 3 BP: 98/52 HR: 73

All scenarios also included learning objectives for non-technical skills

*BiPAP* bilevel positive airway pressure, *BP* blood-pressure, *CPR* cardiopulmonary resuscitation, *CPAP* continuous positive airway pressure, *E* Eye response, *GCS* Glasgow coma scale, *HR* heart rate, *ICU* intensive care unit, *M* motor response, *RSI* rapid sequence induction, *V* verbal response

We tried to avoid exposing the same crew members to similar scenarios during the study period: we ensured that at least the physicians were exposed to different scenarios every time they participated. To ensure this, a coded list of participants for each scenario was kept by the facilitator. If the pilot and the HCM were exposed to the same scenario, the facilitator would alter the context and basic physiological setting and development slightly to allow some variation whilst still adhering to the learning objectives. Hence, each participating crew member was exposed to as many different scenarios as possible during the study period.

We used basic manikins that could be ventilated and intubated. The scenarios were designed to take place in realistic physical settings, for example outdoors, in the helicopter cabin or in a staircase. In accordance with established models of training [10], the equipment used was familiar to the team: monitors, ventilators, and other medical equipment were taken from the helicopter by the crew and replaced immediately after training. The training took place either indoors or outdoors in the immediate vicinity of the HEMS base to minimize any time-delay if the training was interrupted by a tasking.

The day before the scheduled training, the on-call crews received an individual email with the standard operating procedures (SOPs) related to the planned scenario. If a medical observer was present on the base on training day, this person was often enrolled in the scenario, with a role according to their medical background. This protocol is similar to the one used by London Air Ambulance [10].

All simulations started with a short briefing to set the scene, before the crews were taken to the manikin or the scene of the simulation. The facilitator would then verbally give a description of the patient, and provide physiological parameters like blood pressure, heart rate, oxygen saturation when the crews applied appropriate monitoring equipment. Details of pathology, anatomy, and pathophysiology as well as other relevant information, the team would need in the scenario were given in due course as the team performed their assessment of the patient. After completion of the scenario, the team and the facilitator replaced all equipment in readiness for the next mission. Finally, the facilitator led a structured debriefing with the crew to highlight learning points from the simulation [13]. During the scenario and the debriefing, there was a focus on both technical and non-technical skills. If training was interrupted by a real mission, the simulation was aborted, but the opportunity was given to continue the simulation, or debrief, after the mission. All participants are accustomed to simulation within their own field (medicine or aviation). At the end of all debriefing, the crews were encouraged to give feedback and raise any issues related to the training at any time, either in the group setting or individually.

A HEMS physician from the base facilitated the simulations, which could be conducted at any time between 9 am and 3 pm. The facilitator had been trained on a 3-day facilitator course [14]. Except for two occasions, all simulations were facilitated by the same person (PB) to ensure regularity and consistency in content and style of simulation.

### Data collection

A questionnaire was developed to capture the participants’ evaluation of the simulation-based training. An initial version was drafted by two of the authors (PB and SS). The draft questionnaire was reviewed by a representative from each role: a pilot, an HCM, and a physician. Using their input, the questionnaire was modified to optimize and clarify the answer options. This modified version was reviewed by two HEMS crews from the OUH HEMS base to ensure that the questionnaire was clear and comprehensible. The questionnaire was written and presented to the crews in Norwegian. A translated version of the questionnaire is available (Additional file 1). The questionnaire consisted of 14 questions, to be answered on a 7-point Likert scale [15]. The score ranged from 1 to 7 where 1 equaled *I strongly agree* and 7 equaled *I strongly disagree*.

All participating crew members were asked to rate their experience with the simulation and their attitudes to simulation-based training after the completion of each simulation. The questionnaire was either completed immediately after the debrief in front of the facilitator or later the same day and then collected by the facilitator. The responses were anonymized and recorded in an Excel spreadsheet (Microsoft Corp, Redmond, WA, USA) along with other data from the simulation.

The facilitator manually recorded the time taken (to the nearest minute) in each of the four distinct phases of the simulation: time the facilitator spent on preparing the scenario for the simulation; time the crew spent performing the actual simulation; time spent cleaning up and making all the equipment mission-ready; and time spent in debrief. The time of the day of the simulation was also recorded.

### Statistical analysis

Continuous data was summarized using median (quartiles), and categorical data as numbers (percentages). Questionnaire responses were presented graphically using standard bar charts. Data was analyzed using SPSS (IBM Corp. SPSS Statistics for Windows, Version 25, IBM Corporation, Armonk, NY, USA) and R 3.11 [16].

## Results

A total of 44 individual simulations were conducted with a total of 15 physicians, 12 HCM, and 15 pilots participating. The commonest reason for not doing performing a simulation with a HEMS crew was a conflicting live mission or the need for rest after missions during the preceding night. Twenty-two (50%) of the simulations were initiated before 12 pm. On one occasion, the crew opted out of a simulation but agreed to do a “talk through” of a scenario. This session is not included in the study, as no feedback questionnaire was completed. In 11 of the 39 weeks, both helicopter crews on call performed simulation-based training on the same day.

In four (9%) of the simulations, the pilot opted out from the training due to other flight operations related tasks. All other simulations were conducted by the whole HEMS team. One simulation was aborted due to the crew being tasked to a mission during the simulation, but the simulation was subsequently resumed and finished. Four debriefs were interrupted by missions for the crew, and were completed after the mission.

In Table 2, the median (quartiles) time consumption for a simulation training session for the on-call crew and the facilitator is presented, as is the time spent in each of the four phases of the simulation.

**Table 2:** Time consumption for simulation training sessions for facilitator and HEMS crew

Task	People involved	Time used in minutes, median (quartiles)
Preparation of scenario	Facilitator only	10 (5-11)
Scenario simulation	HEMS crew + facilitator	20 (19-26)
Cleaning up after scenario	HEMS crew + facilitator	7 (6-10)
Debrief	HEMS crew + facilitator	35 (30-40)
Total time consumption for on-call HEMS crew	HEMS crew	65 (59-73)
Total time consumption for facilitator	Facilitator only	75 (64-88)

Of the 44 groups of questionnaires handed out after the simulations, one group was missing responses for all crew members and in one group the physician’s response was missing. This left 42 (95%) complete sets of questionnaires available for further analysis.

In Table 3, the participant’s evaluation of the training is presented. The median score on the Likert scale regarding the relevance of the training was 1, range (1-3). Almost all participants (98.4%) used the two most positive categories. For the full training concept, the median score was also 1, range (1-2) and all participants used the two most positive categories. For all other questions on the questionnaire, the median score for participants of all three professional groups provided responses in one of the two most positive categories.

**Table 3:** Median scores (with quartiles) from the participant’s evaluation of the training on a Likert scale

Questions for participants	Physician,median (quartiles)	HCM, median (quartiles)	Pilot, median (quartiles)
1. There was enough time scheduled for the training	1 (1-1)	1 (1-2)	1 (1-2)
2. I felt that the training interrupted my on-call duties	7 (6-7)	6 (5-7)	7 (6-7)
3. There was enough equipment to make the training realistic	2 (1-3)	2 (1.5-3)	2 (1-3)
4. I felt comfortable with the way the training was organized	1 (1-1)	1 (1-2)	1.5 (1-2)
5. I felt uncomfortable when exposing my skills and competencies during the training	6 (6-7)	7 (6-7)	7 (6-7)
6. Simulation is a realistic way of training	2 (1-2)	2 (1-2)	2 (1-2)
7. The topic for the training is relevant to this kind of training	1 (1-1)	1 (1-1.25)	1 (1-2)
8. The clinical aspects of the scenario were good (physician) This type of training is useful for HEMS crew members (HCM) This type of training is useful for HEMS pilots (pilot)	1 (1-1.5)	1 (1-1)	1 (1-1)
9. The scenario relied on procedures that we have already practiced	1 (1-1.5)	1 (1-2)	2 (1-2)
10. The topic for the scenario training seemed relevant for the profile of missions on the base	1 (1-2)	1 (1-2)	1 (1-2)
11. It was useful for me with feedback after the training	1 (1-1)	1 (1-1)	1 (1-2)
12. There was enough time scheduled for feedback after the training	1 (1-2)	1 (1-2)	1 (1-2)
13. It was easy to motivate myself to do this form of training	1 (1-2)	1 (1-2)	1 (1-2)
14. I am positive to this form of training	1 (1-1)	1 (1-1)	1 (1-1)

The score ranged from 1 to 7 where 1 equaled *I strongly agree* and 7 equaled *I strongly disagree*

*HEMS* helicopter emergency medical service, *HCM* HEMS crew member

## Discussion

In this study, we found that it was feasible to introduce an in situ simulation concept for the on-call HEMS crews at a base with a high workload. The training took a short time to carry out and was well received by all crew members. The simulations included training in skills, procedures, and teamwork. We aimed to run scenarios in familiar settings so that the crews were able to identify themselves with the situation. We found that this form for training was not seen as disruptive to on-call work and crews found that the time devoted was sufficient. Feedback also showed that crews found it easy to motivate themselves to participate in this form of training.

In situ simulation can be perceived as an additional strain on the on-call crews, and successful integration with the workflow can pose a challenge. However, in our study, most participants reported that such training did not disrupt their non-clinical duties whilst on call. A positive attitude may play an important role in the successful integration of in situ simulation, as mentioned in the paper by Spurr et al. [17]. A possible additional positive contribution to the success of this training was having a dedicated facilitator with knowledge of the local context, which made it easier to adapt and tailor the simulation to the actual crew and maybe more importantly, to sense when and how the simulation training could be undertaken to cause the least stress to the crew. Another possible success factor might be that the crew, in order to minimize time-wasting, went straight from the HEMS base resting area to the brief for simulation and then straight into the simulation itself. We chose a basic manikin, which in a recent study was found to be as effective as a high fidelity manikin in inducing participant self-reported engagement and learning [18].

There are other potential benefits in favor of low fidelity in situ simulation. Costs are lower: there are no travel expenses, clinical equipment is already in place and readily available for use in the training, and the need to replace personnel participating in the training is attenuated since the participating on-call crew is already present. Dotson et al. found reduced training costs with a high-fidelity air medical simulator [19]. The setup by Dotson included running an advanced and expensive manikin simulation in the helicopter with a rotor turning on the ground. Incurring the costs of helicopter use might not be necessary to induce realism and immersion for simulation in HEMS services.

The participants’ positive attitude towards the training at Kirkpatrick’s level one, (learning evaluation on the reaction level [20]), merely shows that the training was successfully received by the crews. This evaluation is however important to ensure that the concept is seen as useful and that the level of difficulty matches the need of the participants. We planned the training to involve the full HEMS team and included learning objectives related to their skills. This had the potential to improve the interaction within the crew and thus their behavior as team members, but this was not evaluated in this feasibility study. We did, however, observe a possible effect in the organization that the collected data failed to capture: crews’ experiences of the simulations did in some cases lead to changes in standard operating procedures, which can be described as learning on an organizational level [20].

In the simulations, we used a facilitator familiar with both the local procedures and the crews. We believe that this maximizes opportunities to tailor not only the scenario but also the debrief to local training needs.

Our study has some limitations. First, only one HEMS base participated in the study. This limits the generalizability of our findings and the study should be repeated in a larger cohort including more bases to explore if there are other factors influencing implementation that we did not identify in this study. Second, almost all simulations were led by one instructor from the OUH HEMS base. While this was done to reduce variability, we cannot tell whether a larger group of instructors would influence the training and its implementation and success due to individual preferences among the instructors, for example, in when to initiate training. Third, all participants were aware that the training was part of a project of interest for the instructor. This might have induced the crews to answer the questionnaire more positively especially when completed in the vicinity of the facilitator. Fourth, the questionnaire was developed solely for this HEMS setting and has not been validated. This could potentially limit some of the conclusions from the questionnaire. However, limiting the study to only one HEMS base and only one instructor can also be considered a strength in that it was possible to ensure that no participants were exposed to identical scenarios more than once and thereby bored or under-stimulated by to the training.

Future research should be performed to evaluate implementation of in situ simulation in HEMS services and to what extent this training can be shown to improve teamwork and clinical practice.

## Conclusion

We found that in situ simulation training for on-call crews at a busy HEMS base is feasible and can be done with a limited investment of time and resources. The participating crews reported high levels of satisfaction with the training, its organization, and the time devoted to it.

## Supplementary information

**Additional file 1.**

## Data Availability

The dataset used during the current study are available from the corresponding author on reasonable request.

## Abbreviations

BiPAP: Bilevel positive airway pressure
BP: Blood-pressure
CPAP: Continuous positive airway pressure
CRM: Crew resource management
CPR: Cardio pulmonary resuscitation
E: Eye response
EASA: European Aviation Safety Agency
ECMO: Extra corporeal membrane oxygenation
EMS: Emergency medical services
GCS: Glasgow coma scale
HCM: HEMS crew member
HEMS: Helicopter emergency medical service
HR: Heart rate
IABP: Intra-aortic balloon pump
ICU: Intensive care unit
M: Motor response
NIV: Non-invasive ventilation
OUH: Oslo University Hospital
RSI: Rapid sequence induction
SOPs: Standard operating procedures
V: Verbal response
