# Supplementary material for: In situ simulation training in helicopter emergency medical services: feasible for on-call crews?
Source: Adv Simul (Lond). 2020 Jun 15;5:7. doi: 10.1186/s41077-020-00126-0 (PMC7294664; doi:10.1186/s41077-020-00126-0)
Supplement: Supplementary file 1 — Additional file 1. [file 41077_2020_126_MOESM1_ESM.docx]

Appendix
Questionnaire translated from Norwegian language:

1. There was scheduled enough time for the training
2. I felt that the training was disturbing for my on call duties
3. There was enough equipment to make the training realistic
4. I felt comfortable in the way the training was organised
5. I felt uncomfortable when exposing my skills and competencies during the training
6. Simulation is a realistic way of training
7. The topic for the training is relevant for this kind of training
8. This type of training is useful for HEMS crew members
   This type of training is useful for HEMS pilots
   The clinical aspects of the scenario was good
9. The scenario relied on procedures that we have already practised
10. The topic for the scenario training seemed relevant for profile of missions on the base
11. It was useful for me with feedback after the training
12. There was enough time scheduled for feedback after the training
13. It was easy to motivate myself to do this form for training
14. I am positive to this form of training

All questionnaires were equal for physician, pilot and HCM apart from question no 8 which was tailored to each group.

Answers:
1 = Completely agree

2= Almost agree

3= Partly agree

4= Neutral

5= Partly disagree

6= Almost disagree

7= Completely disagree
